# Comparative analysis of the effectiveness difference of SARS-COV-2 mRNA vaccine in different populations in the real world: A review

**DOI:** 10.1097/MD.0000000000034805

**Published:** 2023-08-25

**Authors:** Sihui Cai, Chunyan Chang, Xiuhong Zhang, Weizhen Qiao

**Affiliations:** a Department of Laboratory Medicine, The Affiliated Wuxi People’s Hospital of Nanjing Medical University, Wuxi People’s Hospital, Wuxi Medical Center, Nanjing Medical University, Wuxi, China; b Department of Pharmacy, The Affiliated Wuxi People’s Hospital of Nanjing Medical University, Wuxi, China.

**Keywords:** difference, effectiveness, mRNA vaccine, SARS-CoV-2

## Abstract

Coronavirus disease 2019 (COVID-19) has ravaged the world since December 2019. Up to now, it is still prevalent around the world. Vaccines are an important means to prevent the spread of COVID-19 and reduce severe disease and mortality. Currently, different types of novel coronavirus vaccines are still being developed and improved, and the relevant vaccines that have been approved for marketing have been widely vaccinated around the world. As vaccination coverage continues to grow, concerns about the efficacy and safety of vaccines after real-world use have grown. Some clinical studies have shown that vaccine effectiveness is closely related to antibody response after vaccination. Among them, the advantages of COVID-19 messenger ribonucleic acid (mRNA) vaccine, such as better adaptability to variant strains and better immune response ability, have attracted great attention. However, different populations with different genders, ages, previous COVID-19 infection history, underlying diseases and treatments will show different antibody responses after mRNA vaccination, which will affect the protection of the vaccine. Based on this, this paper reviews the reports related severe acute respiratory syndrome Coronavirus 2 mRNA vaccines, and summarizes the effectiveness of vaccines in different populations and different disease states and looked forward to the precise vaccination strategy of the vaccine in the future.

## 1. Introduction

The Coronavirus disease 2019 (COVID-19), caused by the severe acute respiratory syndrome Coronavirus 2 (SARS-CoV-2), is still prevalent all over the world.^[1]^ Given that there is no specific treatment drug for COVID-19 and SARS-CoV-2 spreads very fast, the prevention and control of this disease depend on vaccination. Accelerating vaccination and increasing SARS-CoV-2 vaccine coverage may be the most effective means at present. Therefore, the effectiveness of the SARS-CoV-2 vaccine is a public concern, which is the biggest factor affecting the willingness of vaccination.^[2–6]^ The messenger ribonucleic acid (mRNA) vaccine research platform has been extensively studied for the treatment of a variety of infectious diseases and tumors over the past decades, and has been proven to have good safety and strong immunogenicity, however, no mRNA vaccine has ever been approved for clinical use before the COVID-19 pandemic.^[7–9]^ Thus, there are few reports on the effectiveness of mRNA vaccines after inoculation. The large-scale application of the SARS-CoV-2 mRNA vaccines, mRNA-1273, and BNT162b2, developed by Moderna and Pfizer/BioNTech respectively, makes it possible to analyze the effectiveness of mRNA vaccine in the real world. This article reviews the effectiveness of SARS-CoV-2 mRNA vaccine after vaccination, focusing on comparing the level and duration of immunogenicity response indicators stimulated by mRNA vaccine in different vaccinated populations in the real world, and looks forward to the precise vaccination strategy in different populations.

## 2. Research status of SARS-COV-2 mRNA vaccine

According to the statistics of World Health Organization (WHO), as of Mar 2023, 183 vaccines are under clinical research, and another 199 vaccines are under pre-clinical trial worldwide.^[10]^ In the background of the global pandemic of COVID-19, a number of vaccines have been authorized for use, including 11 COVID-19 vaccines approved by WHO for emergency marketing worldwide. There are 2 mRNA vaccines, mRNA-1273 and BNT162b2. Both vaccines are nucleoside modified mRNA vaccines encapsulated with lipid nanoparticles, and developed against the Spike protein of SARS-CoV-2.^[11]^ The mRNA vaccine is a single strand RNA vaccine, which obtains the genetic information by transcription and synthesizes gene fragments encoding specific pathogen antigen protein in vitro. After vaccination, the gene fragments are introduced into the body and expresses as corresponding antigen proteins, which can prevent diseases by inducing the immune system response to the antigen protein.^[12]^ Compared with traditional non-mRNA vaccines, mRNA vaccines usually have several advantages. First, as a noninfectious or nonintegrated technology, the development of mRNA vaccines has a relatively safety without the potential risk of infection or embedded mutation. Second, nucleoside modification in mRNA vaccines can improve the stability and translation ability of mRNA. Moreover, lipid nanoparticles have been proved to be effective carriers for delivering mRNA in vivo, which is conducive to rapid uptake and expression of host cells, and ultimately stimulate the body to produce a strong specific immune response. Third, an mRNA candidate vaccine against the target virus can be developed with known gene sequence information in a short time on the mRNA vaccine technology platform, which is highly versatile.^[13,14]^ These advantages are also an important reason why the SARS-CoV-2 mRNA vaccine has been approved by WHO and included in the emergency use list. Studies on antibody responses to 2 commercially available mRNA vaccines in healthy people have shown that BNT162b2 and mRNA-1273 have similar effects in inducing various anti-SARS-CoV-2 antibodies, and both showed efficacy against variant virus strains. For example, Moderna mRNA-1273 vaccine was effective against the Alpha (B.1.1.7), Beta (B.1.351-v1, B.1.351-v2, and B.1.351-v3), Delta (B.1.617.2), and Gamma (P.1) variants of COVID-19. Pfizer-BioNTech BNT162b2 vaccine was effective against Alpha (B.1.1.7) and Beta (B.1.351-V) variants.^[15–17]^ Also, relevant studies have pointed out the effectiveness of the BNT162b2 vaccine and mRNA-1273 vaccine in preventing infection and reducing hospitalization rates caused by Omicron (B.1.1.529) variant which is currently prevalent worldwide.^[18,19]^ At present, 43 of the 173 vaccines in clinical stage are mRNA vaccines, including 16 in stage I, 8 in stage I/II, 4 in stage II, 5 in stage II/III, 7 in stage III, and 3 in stage IV. Table 1 lists the details of COVID-19 mRNA vaccines in phase I-IV.

**Table 1:** Overview of COVID-19 mRNA vaccines in stage I to IV as of March 2023.

Phase	Vaccine name	Number of doses	Schedule	Developers	Trial registries
IV	mRNA-1273	2	D 0 + 28	Moderna + National Institute of Allergy and Infectious Diseases (NIAID)	NCT04760132
	BNT162b2	2	D 0 + 21	Pfizer/BioNTech + Fosun Pharma	NCT04760132
	mRNA-1273.351.	3	D 0 or D 0 + 28	Moderna + National Institute of Allergy and Infectious Diseases (NIAID)	EUCTR2021-000930-32
III	CVnCoV	2	D 0 + 28	CureVac AG	NCT04674189
	ARCoV	2	D 0 + 14 or D 0 + 28	Academy of Military Science (AMS), Walvax Biotechnology and Suzhou Abogen Biosciences	NCT04847102
	PTX-COVID19-B	2	D 0 + 28	Providence Therapeutics	NCT05534035
	ARCT-154	2	D 0 + 28	Arcturus Therapeutics, Inc.	ISRCTN15779782
	LVRNA009	2	D 0 + 28	AIM Vaccine and Liverna Therapeutics	NCT05428592
	mRNA-1273.214	2	D 0 + 55	ModernaTX	NCT05436834
	SW-BIC-213	2	TBD	Shanghai East Hospital and Stemirna Therapeutics	NCT05580159
II/III	DS-5670a	2	NR	Daiichi Sankyo Co., Ltd.	JPRN-jRCT2071210106
	HDT-301	2	D 0 + 28	SENAI CIMATEC	NCT05542693
	mRNA-1273.211.	1	D 0	ModernaTX, Inc.	NCT04927065
	mRNA-1273.529	1	D 0	ModernaTX, Inc.	NCT05249829
	mRNA GEMCOVAC-19	2	D 0 + 28	Gennova Biopharmaceuticals Limited	CTRI/2022/04/041880
II	ARCT-021	NR	NR	Arcturus Therapeutics	NCT04668339
	MRT5500	2	D 0 + 21	Sanofi Pasteur and Translate Bio	NCT04798027
	SYS6006	2	D 0 + 21	CSPC ZhongQi Pharmaceutical Technology Co., Ltd.	NCT05439824
	ChulaCov19	2	D 0 + 21	Chulalongkorn University	NCT05605470
I/II	GLB-COV2-043	1	D 0	GreenLight Biosciences, Inc.	NCT05602961
	JCXH-221	1	D 0	Immorna Biotherapeutics, Inc.	NCT05743335
	EXG-5003	1	D 0	Elixirgen Therapeutics, Inc	NCT04863131
	ARCT-165	2	D 0 + 29	Arcturus Therapeutics, Inc.	NCT05037097
	ARCT-021 mRNA Vaccine	2	D 0 + 30	Arcturus Therapeutics, Inc.	NCT05037097
	EG-COVID	3	D 0 + 21 + 42	EyeGene Inc.	NCT05188469
	AAHI-SC2 and AAHI-SC3	1	D 0	ImmunityBio, Inc.	NCT05370040
	mRNA-1073	2	D 0	ModernaTX	NCT05375838
I	LNP-nCoVsaRNA	2	NR	Imperial College London	ISRCTN17072692
	CoV2 SAM (LNP) vaccine	2	D 0 + 30	GlaxoSmithKline	NCT04758962
	mRNA-1283	2	D 0 + 28	ModernaTX, Inc.	NCT04813796
	COReNAPCIN	1	D 0	ReNAP Technology	IRCT20230131057293N1
	LNP-nCOV saRNA-02 vaccine	2	D 0 + 28	MRC/UVRI and LSHTM Uganda Research Unit	NCT04934111
	HDT-301 vaccine	1–2	D 0 +/− 56	HDT Bio	NCT05132907
	VLPCOV-01	2	NR	VLP Therapeutics Japan GK	jRCT2071210067
	CV2CoV	1	D 0	CureVac AG	NCT05260437
	MIPSCo-mRNA-RBD-1	1	D 0	University of Melbourne	NCT05272605
	A Lyophilized COVID-19 mRNA Vaccine	1	D 0	Jiangsu Rec-Biotechnology Co., Ltd.	NCT05351450
	Lyophilized COVID-19 mRNA Vaccine	1	D 0	Wuhan Recogen Biotechnology Co., Ltd.	NCT05366296
	RQ3013	1	D 0	Walvax Biotechnology; Shanghai RNACure Biopharma	NCT05396573
	RVM-V001	1	D 0	RVAC Medicines	NCT05420077
	ABO1009-DP	1	D 0	Suzhou Abogen Biosciences Co., Ltd.	NCT05433194
	samRNA Vaccines	2	D 0 + 28	Gritstone bio, Inc.	NCT05435027
	CV0501	1	D 0	GlaxoSmithKline	NCT05477186

LNP = lipid nanoparticles, mRNA = messenger ribonucleic acid, NR = not reported, RBD = receptor binding domain, TBD = to be defined.

## 3. Indicators for effectiveness analysis of mRNA vaccine

The effectiveness is a core indicator for evaluating a vaccine. In animal experiments, the effectiveness of the mRNA vaccine is usually evaluated by detecting the serum antibody level of the vaccinated animals.^[20–23]^ In the clinical trial phase, protective efficacy, the gold standard for effectiveness evaluation, is used to evaluate the effectiveness of different COVID-19 vaccines based on disease status. The protective efficacy was evaluated by calculating the vaccine protection rate: protection rate = 1 − incidence in the vaccine group/incidence in the placebo group.^[24]^ In the phase III clinical trial of Pfizer BNT162b2, 43,448 people over 16 years old were recruited globally, and were divided into vaccine group and placebo group according to 1:1. Two doses of BNT162b2 or placebo were given 21 days apart. Finally, there were 170 confirmed cases of COVID-19, including 8 cases from the vaccine group and 162 cases from the placebo group, resulting in a calculated protective efficacy of 95.1% for BNT162b2. According to the trial, BNT162b2 could show good protective efficacy for at least half a year after 2 doses of complete inoculation.^[25,26]^ The phase III clinical trial of mRNA-1273 included 30,420 subjects, who were randomly divided into vaccine group and placebo group at the ratio of 1:1, and were vaccinated interval of 28 days. Finally, 196 cases were infected, among which 11 cases in the vaccine group and 185 cases in the placebo group. Thus, the protective efficacy of the vaccine was 94.1%.^[27]^ Although protective efficacy is the gold standard for evaluating the effectiveness of a vaccine, it often requires a very large sample size in clinical trials, which consumes huge manpower and material resources. However, immunogenicity data, such as geometric mean titer of antibody, serum conversion rate, IgG antibody titer or the response level of other specific antibodies to the receptor-binding domain, are simple and intuitive, which still reflect the effect of vaccine to a certain extent.^[24]^ In addition, several modeling and efficacy trials have shown that neutralizing antibody levels could predict protection against infection or severe disease. There was also a close correlation between the reported efficacy of several COVID-19 vaccine trials and neutralizing antibody levels.^[28–30]^ Therefore, immunogenicity data were used as a substitute for analysis on effectiveness of vaccination in many published studies. At present, the global COVID-19 vaccination work is continuing to advance. It is crucial for subsequent guidance in formulating scientific vaccination strategies to understand the differences in the effectiveness of vaccination among different groups.

## 4. The effectiveness difference of populations in different states after vaccination with SARS-COV-2 mRNA vaccine in the real world

### 4.1. Gender

Gender differences are mainly physiological. Ruggieri et al found that women have stronger immune response compared with men.^[31]^ Vassilaki et al reported an analysis of antibody response to SARS-CoV-2 mRNA vaccine in Greek medical staff in September 2021. The study monitored the level of IgG antibody to the receptor binding domain (RBD) of anti-SARS-CoV-2 S protein in 1643 (533 males/1110 females; median, 49 years) volunteers after receiving 2 doses of vaccine. None of these volunteers had a previous infection history of SARS-CoV-2. Intravenous blood testing showed 1636 (99.6%) volunteers turned positive after the second vaccination. The antibody titer of women (median 1594, interquartile range [IQR] = 875–2548) was 1.2 times higher than that of men (median 1292, IQR = 671.9–2188) (*P* = .00003).^[32]^ Paal et al retrospectively assessed anti-SARS-CoV-2 S protein antibody levels in 179 maintenance hemodialysis patients (114 males and 65 females) 3 to 6 weeks after the second dose of mRNA vaccine. The median antibody titer of SARS-CoV-2 in females was 302 (IQR = 82.5–799.5), and that in males was 233 (IQR = 42–643). Multivariate analysis showed that women had a higher level of vaccine response among maintenance hemodialysis patients (*P* = .006).^[33]^ Similarly, in Terpos et al cohort study of 255 health workers (92 men and 163 women; median, 49 years) and 112 vaccinated octogenarians (51 men and 61 women; median, 85 years), it was found that, regardless of age cohort, women had higher anti-Spike-RBD immune titers than men at the time of the second dose of BNT162b2 vaccine. Also, on days 22 and 50 after vaccination, women in octogenarians had higher neutralizing antibody titers than men (*P* < .05).^[34]^

### 4.2. Age

For individuals, the function of immune system will change with age. Thus, age is also a relatively important factor affecting vaccine immune response.^[35–37]^ Jabal et al described the trend of anti-S protein IgG levels with age in 514 Israeli medical personnel on day 21 after the first dose of BNT162b2 mRNA vaccine. The results showed that IgG antibodies were detected in 475 (92%) of the vaccinated population, with a geometric mean concentration of 68.6 AU/mL. However, the 39 non-responders to the first dose of vaccine were older (median, 57 years), which was significantly different from the responders (median, 45 years) (*P* < .001). Moreover, antibody titers were found to decrease with age in antibody responders (*P* < .001). This trend still existed even when excluding previously infected respondents (*P* < .001).^[38]^ In addition, a similar study by Tsatsakis et al also showed that an older age was associated with the lower the antibody titers (*P* < .001).^[39]^ However, Watanabe et al conducted a multifactorial antibody titer study in 86 healthcare workers (median, 29 years) in Rome who received 2 doses of COVID-19 mRNA vaccine in 2021. The blood samples were taken for analysis of antibodies against the S protein. Multivariate analysis of the study showed that antibody titer was independent of age (*P* > .05).^[40]^ The differences in the above analysis results might be related to different research methods, the number of people included in the study, and different quantitative indicators. In addition, the subjects in Watanabe et al study were young and had a small age span, which might lead to incomplete analysis of the age factor results.

### 4.3. Race or ethnic group

Several reports had revealed ethnic differences in cancer incidence and mortality, as well as individual growth and associated metabolism.^[41,42]^ Some reports investigated the relationship between ethnic differences in hospitalization rates, mortality and prognosis of COVID-19 during the COVID-19 epidemic.^[43–45]^ The differences in COVID-19 vaccine among different ethnic groups are also of interest. Cai et al reported that the vaccine efficacy of black or African Americans was higher than that of white people, and the vaccine efficacy of black or African American and white participants was 95.37% and 89.81%, respectively.^[46]^ In a phase III trial of Pfizer BNT162b2, 37,706 participants responded at least 2 months after receiving 2 doses of the vaccine. Of those participants, 83% were white, 9% were black or African American, 4.2% were Asian, and 28% were Hispanic or Latino. The report showed that vaccine efficacy in subgroups defined by race and ethnicity was broadly consistent with that observed in the overall population. The results were 95.2% for whites, 100% for blacks or African Americans, 94.4% for Hispanics or Latinos, and 89.3% for all other races after 2 doses of BNT162b2 vaccine.^[25]^

### 4.4. Previous SARS-CoV-2 infection history

Many studies reported that the antibody titer of people with a history of SARS-CoV-2 infection increased more significantly than that without SARS-CoV-2 infection after receiving a single dose of vaccine, and a single dose of vaccine may be sufficient for them. Racine et al conducted a longitudinal study to compare the antibody response of 68 patients vaccinated with BNT162b2 vaccine (19 patients previously infected with SARS-CoV-2, RecoVax group; 49 patients never infected with SARS-CoV-2, NaiveVax group). In the RecoVax group, the median time, from the onset of COVID-19 symptoms to the first vaccination (D1), was 262 days (IQR 102–275). Before vaccination, there were detectable antibodies against SARS-CoV-2 S protein, and after D1, the antibody level in this population increased from an average of 47.45 U/mL (IQR19.59–148.7) to higher than the upper limit of detection (>2500 U/mL). It remained at this level for about 6 months after D1. The antibody level in the NaiveVax group gradually increased to an average of 37.77 U/mL (IQR 12.93–80.45) before the second dose of vaccine (D2) after D1. Two weeks after D2, the average antibody was 2177 U/mL (IQR 1605–>2500 U/mL). However, about 6 months after D2, the antibody level dropped to an average of 720 U/mL (IQR 565–1269). Although the total antibody level against S protein increased after D2, the antibody level in NaiveVax group was always lower than that in RecoVax group (*P* <.001 at all-time points). These data indicated that people previously infected with SARS-CoV-2 had a stronger antibody response after vaccination, and a single dose of vaccine caused a strong enough antibody immune response in the RecoVax group.^[47]^ Similarly, Fraley et al compared the antibody responses of the group without SARS-CoV-2 infection history and the group with SARS-CoV-2 infection 30 to 60 days before D1 after BNT162b2 vaccination. Three weeks after the initial inoculation, antibody titers to the 3 S subunit antigens (S1, S2, RBD) were significantly higher in both groups than the baseline. Moreover, the antibody titers of all S antigens in the infection group were significantly higher than those in the uninfected group (*P* < .001). 4 weeks after the second inoculation, the titers of the 3 S subunit proteins antibody in the uninfected group were significantly higher than those at the third week. The antibody levels of S1 and S2 were significantly higher in the infected group, but the RBD antibody level did not change significantly. The titers of S1 and RBD antibodies in the infected group at the 3rd week were similar to those in the uninfected group at the 7th week, but the levels of S2 antibody in the infected group at the 3rd and 7th weeks were significantly higher than those in the uninfected group (*P* < .001). The study also found that although the N protein is contained in the SARS-CoV-2, only the S protein is the vaccine targeted antigen. So higher level of N protein antibody was found only in the infection group. There was no significant change in N protein antibody levels after vaccination in both groups. The research showed that the population infected with SARS-CoV-2 had a higher antibody level after single dose vaccination, roughly equivalent to those observed in uninfected population after 2 doses of vaccination.^[48]^

### 4.5. People with immune deficiency caused by underlying diseases

The underlying disease is also one of the factors potentially affecting the protective efficacy of vaccines. In general, people with autoimmune diseases have about 1.7 times the risk of getting infected by the virus than the general healthy people. Moreover, when the vaccinators suffered from obesity, hypertension, diabetes, chronic renal insufficiency, autoimmune suppression and other diseases, these diseases affected the immune function of the human body, thereby affecting the protective efficacy of the vaccine, resulting in different antibody responses to the vaccine.^[24,35]^ Watanabe et al pointed out that obesity (correlation coefficient *R* = −0.324, *P* = .004) was associated with lower vaccine response antibody level by multivariate antibody titers analysis for 2 doses of SARS-CoV-2 mRNA vaccine. The antibody level was lower in hypertensive population than that in normotensive population (*P* = .001). Compared with people with normal blood lipids, those with abnormal blood lipids had lower antibody levels (*P* = .005).^[40]^ In a word, obesity, hypertension and dyslipidemia were related to low antibody titers. In addition, Havlin et al studied the antibody response to SARS-CoV-2 mRNA vaccine in lung transplant recipients with low immune function. In this study, lung transplant recipients who vaccinated with 2 doses of BNT162b2 mRNA vaccine did not produce IgG against S protein.^[49]^ Itzhaki et al studied the antibody response of BNT162b2 mRNA vaccine in heart transplant recipients. The results showed that about half of heart transplant recipients did not produce S-IgG after 2 doses of vaccine.^[50]^ Rozen et al analyzed that in renal transplant recipients. Among 308 recipients, only 112 (36.4%) showed positive anti-SARS-CoV-2 S protein antibody 2 to 4 weeks after the second dose of BNT162b2 mRNA vaccine.^[51]^ The conclusions of these studies indicated that organ transplant recipients had an insufficient antibody response to SARS-CoV-2 mRNA vaccine, and that the level of immunosuppression was an important factor in this response. In addition, Simon et al compared antibody responses to mRNA vaccination in a population receiving hemodialysis (dialysis for at least 3 months) with those in a healthy population, which showed that antibody levels detected 21 days after 2 doses of vaccine. The antibody titer in the hemodialysis group (median 171 U/mL) was significantly lower than that in the healthy group (median 2500 U/mL), and there was a delay in vaccine antibody response.^[52]^ Ali et al studied antibody responses to COVID-19 mRNA vaccines in patients with multiple sclerosis who received B-cell depletion therapy (BCTD). The study found that the body humoral immune response was impaired by BCTD treatment, and the positive rate of antibodies was only 36.4% (100% in the control population) (*P* < .0001). Moreover, the time interval between the last infusion of BCTD treatment and the first injection of vaccine was relatively short, which was related to the negative response of vaccine antibody.^[53]^ While the research of Picchianti et al showed for the first time that the specific antibody and specific T cell reaction against S protein induced by SARS-CoV-2 mRNA vaccine existed in most rheumatoid arthritis patients who took the strategy of suspending immunosuppression during vaccination. In addition, BNT162b2 mRNA vaccine was safe and disease remained stable in such arthritis patients.^[54]^ Therefore, it was a worthwhile direction to find out the appropriate time to suspend immunosuppressive therapy for these special populations to prevent SARS-CoV-2 infection by vaccine. Human immunodeficiency virus infection will cause a breakdown of the immune system, which theoretically affects the course and effectiveness of vaccination, but people living with human immunodeficiency virus (PLWH) also need an effective vaccine against SARS-CoV-2. Bociąga-Jasik et al investigated 121 PLWH vaccinated against SARS-CoV-2 infection. 86 (71%) of these patients were vaccinated with the mRNA vaccine and the mean values of anti-S IgG and IFN-γ in these populations after vaccination were 298.58 BAU/mL, 3261.35 mIU/mL (BNT162b2); 508.56 BAU/mL, 5666.41 mIU/mL (mRNA-1273) respectively, which was stronger than in patients who received other vaccines (*P* = .000). This study indicates that COVID-19 vaccines were immunogenic and safe in PLWH and that mRNA vaccination, particularly mRNA-1273, was associated with better humoral and cellular responses.^[55]^

### 4.6. Pregnant and lactating women

SARS-CoV-2 infection is more severe in pregnant women than in non-pregnant women. And immunological properties of breast milk also change during lactation. Despite the higher risk and harm associated with SARS-CoV-2 infection, pregnant and lactating women were excluded from any initial COVID-19 vaccine trials. There are significant concerns about the safety of COVID-19 vaccines for pregnant and lactating women. Recently, studies in several countries and regions have shown that COVID-19 mRNA vaccines have a good safety profile during pregnancy, with a lower incidence of major adverse events in pregnant women than in non-pregnant vaccine recipients. Vaccination also does not increase the incidence of adverse perinatal outcomes, including fetal morphologic abnormalities. The Centers for Disease Control and Prevention and the American College of Obstetricians and Gynecologists recommend that lactating patients receive COVID-19 mRNA vaccines, and there is no evidence that breast milk from lactating individuals who have received COVID-19 mRNA vaccines causes harm to lactating infants.^[56–58]^ However, the antibody response in this population after vaccination is also a concern. At present, some prospective cohort studies have reported the response to vaccination in such populations. Charepe et al compared the antibody responses of lactating women and non-lactating women to SARS-CoV-2 mRNA vaccine. The study included a random sample of 24 infected medical staff (14 lactating and 10 non-lactating women). There were no significant differences among these women in age, smoking habits or underlying medical conditions. The antibody levels against the SARS-CoV-2 S protein were tested in blood samples and breast milk taken 1 to 3 weeks after the first and second vaccinations. The results showed that the average antibody level of non-lactating women was higher than that of lactating women after D1, but that of all women was high after the second vaccination. A moderate level of antibodies was also observed in breast milk of lactating mothers, especially 42.9% of breast milk after the second vaccination.^[59]^ Gray et al found that indicators of humoral immune responses caused by SARS-CoV-2 mRNA vaccine in pregnant and lactating women were comparable to those observed in non-pregnant populations.^[60]^ Dagan et al reported a study of the efficacy of BNT162b2 mRNA vaccine in pregnant women with 21,722 participants, indicating that the vaccine had a high protective efficacy in pregnant women, which was similar to the efficacy in the general population.^[61]^

## 5. Suggestion and limitation

COVID-19 has a significant impact on global public health and economic activities. Today, the protective efficacy of vaccines has become the focus of attention as a means to effectively prevent and control the epidemic of the virus. Current clinical studies have shown that the high antibody responses were caused by SARS-CoV-2 mRNA vaccination in normal healthy people. In addition, most studies indicated that the response to SARS-CoV-2 mRNA vaccination in older adults was weaker than that in other adults. The effectiveness of the vaccine was higher in blacks or African Americans than in other races. Moreover, the level of antibodies produced by women was higher than that of men, and there was no statistical difference between pregnant and lactating women and non-lactating women after vaccination, and related antibodies could be detected in milk. However, more follow-up studies were needed to determine whether the antibodies in milk could be used to establish the related immune barrier of infants. For people with special diseases, such as organ transplant recipients or patients needed immunosuppressive therapy, the antibody production rate was lower after the 2 doses of vaccine. Should this special population receive the same dose at the same interval as healthy people, or can the interval be shortened? While it was a research direction to suspense immunosuppressive therapy during vaccination, more subsequent research was needed to find out strategies to guide to effectively prevent SARS-CoV-2 in such populations. Of note, a single dose of SARS-CoV-2 mRNA vaccine in infected population was sufficient to produce antibody levels, which was comparable to 2 doses in uninfected population. Considering the rapid spread of COVID-19 and the unequal distribution of vaccine resources around the world, vaccination strategies could be optimized, and only 1 dose of SARS-CoV-2 mRNA vaccine was given to such populations. At present, the observation time from vaccine development to marketing to vaccination is still short. In particular, SARS-CoV-2 is constantly mutating, vaccines against variants are constantly being developed, and the efficacy of existing vaccines against variants is still under observation. For example, it has been reported that the S protein in mRNA vaccines can increase cardiovascular diseases. In addition to the risk of infection due to reduced immune function, the vaccine may also cause unknown organ damage. Among them, the most representative Pfizer and Moderna mRNA vaccines have also been associated with some mild adverse reactions, including pain at the vaccine injection site, redness and swelling, fever, fatigue, headache, muscle pain, nausea, vomiting, itching, chills and arthralgia, but rarely cause anaphylactic shock. Injection-site reactions are the most common side effects of COVID-19 vaccination. These reactions are mostly mild or moderate, have no serious consequences, and are usually self-limiting without any intervention.^[62–64]^ Recognition of these reactions can play a key role in vaccine strategy, as anxiety about developing a reaction can largely influence willingness to accept a first dose or return a second dose. Therefore, compared with the serious consequences caused by COVID-19 infection, whether these reactions should deter people from vaccination for special populations needs to be comprehensively evaluated. In conclusion, targeted and precise vaccination strategies should be carried out for different populations, so as to provide support for improving the utilization efficiency of vaccines and establishing an effective immune barrier.

MD-D-23-04407

## Author contributions

**Conceptualization:** Weizhen Qiao.

**Writing – original draft:** Sihui Cai, Chunyan Chang.

**Writing – review & editing:** Xiuhong Zhang, Weizhen Qiao.
